# Exploring perceptions of sexuality among youth with physical disabilities in Gweru, Zimbabwe

**DOI:** 10.4102/ajod.v13i0.1363

**Published:** 2024-07-24

**Authors:** Tapson Mashanyare, Tendayi C. Garutsa, Kiran Odhav

**Affiliations:** 1 Department of Sociology, Faculty of Humanities, North-West University, Mmabatho, South Africa

**Keywords:** youths with disability, perceptions, sexuality, socio-cultural perspectives, Gweru, Zimbabwe

## Abstract

**Background:**

Disability and sexuality are topical issues although they are not given much-deserved attention in most societies, and Zimbabwe is not an exception. The socio-cultural stigma associated with disability adversely impacts sexuality and seeps into the social existence of youth with disability. Youths with disability are assumed to be hypersexual or asexual.

**Objectives:**

This article explores the sexuality views and experiences of youth with disability in the context of the negative stigma associated with disability and sexuality in Gweru, Zimbabwe.

**Method:**

Semi-structured interviews and focus group discussions were conducted among 20 Gweru youth (18–35 years old) with physical disabilities and five key informants. Perceptions of youth with disability and socio-cultural assumptions regarding their sexuality were analysed.

**Results:**

The study established that most youths with disability faced social closure in terms of sexuality, with sparse offerings of sexuality education in their families. One sexual education theme that emerged from this study is sexual abstinence. Some suggestions of more open forms of communication on sexuality and disability also emerged although as a minority view.

**Conclusion:**

It was concluded from the study that, most youth feel that they are denied information on sexuality in their families and communities, as they are wrongfully assumed to have no need for it. However, youths with disability do not passively accept the negative perceptions about their sexuality, and they demonstrate their agency in resisting such negative perceptions.

**Contribution:**

The study contributes to knowledge on sexuality and disability among youths with disability in contexts where strong traditional beliefs, myths, and misconceptions exist.

## Introduction

Youths with disabilities are among the most marginalised groups and are likely to experience severe economic, and social disparities compared to youth without disabilities (UNDESA 2018). Access to information on human immunodeficiency virus/acquired immunodeficiency syndrome (HIV/AIDS), sexuality, and reproductive health is usually unavailable to youths with disabilities (Mathabela, Madiba & Modjadji 2024). Article 23 of the Convention on the Rights of Persons with Disabilities (CRPD) implores member countries to provide education and family planning to individuals with disabilities on equal basis with their counterparts without disabilities (Landson 2022). Provision of family planning services to people with disabilities is still a challenge for some African countries because of social norms on disability (Yesgat et al. 2020). In some African societies, people with disabilities are stigmatised and viewed as asexual or hypersexual, while disability is considered a curse on account of negative socio-cultural norms (Karimu 2017; Sande 2019).

Most cultures in Zimbabwe view sexuality as a taboo and an embarrassing subject, which cannot be discussed openly, out of respect. People who discuss sexuality openly are considered wayward and immoral (Peta 2017). There are few chances for youths with disabilities to learn about sexuality from their parents and guardians (Chikate 2020; Mudzimu 2021; Peta 2017). For some youths who manage to go to school, sexuality education is offered in Zimbabwe as part of the school curriculum. However, there is a lack of sexuality education programmes that target out of school youths. Some youth rely on peers and the Internet for sexuality information. Youths without disabilities have more opportunities to learn about sexuality such as in the family, at school, and in the community. Youths with disabilities are denied opportunities to attend community-based programmes on sexual and reproductive health because of assumptions that they do not need such information (Rugoho et al. 2020; Taylor & Abernathy 2022).

Similarly, a study carried out by Chappell (2016) in KwaZulu-Natal, South Africa, reported a lack of parent to child communication on sexuality issues because of social values of respect. Many parents believe that youths with disabilities do not need such education and have anxieties and fears about the disabled youth engaging in sexual activities (Kamaludin et al. 2022; Kok & Akyuz 2015). Access to sexuality education is hindered by several barriers. These include parental beliefs, stereotypes, a lack of opportunity for sexuality training, and fear that sexual information and education may lead to uncontrollable sexual behaviour (Rugoho et al. 2020).

Parental protection of youths with disabilities from any form of sexual experience usually results in their lack of sexual exposure and socialisation. There is a higher likelihood of adverse sexual and reproductive health outcomes for youths with disabilities (Chappell 2016; Frappier 2021; Rugoho et al. 2020).

In some families, there are differences in support for male and female sexuality, whether through sexual services of sex workers (Liddiard 2014) or simply providing information on sexual matters to encourage young males to gain partners. thirdly, such families follow a strict approach of not providing sexuality information to young females and restricting them to explore sexuality with another person but allowing young males the freedom to do so (Rugoho et al. 2020).

Assumptions of passive, docile sexuality, or asexuality, negatively impact sexual autonomy of youths with disabilities (Carew et al. 2020; Chappell 2016; Rohleder 2015; Sakairi 2020). Family socialisation of children with disabilities relates to areas that are off limits in such a discourse: marriage, dating, offspring, and sexuality (Rugoho et al. 2020; Slater et al. 2018). Ultimately, family socialisation erases their sexuality.

The institutionalisation of such assumptions within health service structures effectively excludes youths with disabilities from Sexual and Reproductive Health (SRH) services. Because of the perceived danger of ambiguous sexuality (hyper-sexual and/or asexual) of persons with disabilities, families either strictly monitor their sexuality or ignore it (Holland-Hall & Quint 2017). Sexual reproductive health services are thus impacted by an institutional manifestation of such societal beliefs, with the denial of such services to the youths with disabilities (Rugoho et al. 2020). Such an institutionalisation has mechanisms in place, wherein health workers label people with disabilities who test positive for HIV or have sexually transmitted infections (STIs) as promiscuous (Okello 2018), and this is particularly so for women with disabilities (Lodenious 2020; Rugoho 2019). This stigma results in some people with disabilities not returning to the health facilities, which further exposes them to other health-related complications such as STIs. This article answers the following main research question: what are the factors that hinder dating, sexuality education, marriage, and childbearing experiences for youths with disabilities?

## Methods

Qualitative research methods were used for data collection. Qualitative research enables researchers to capture meanings attached to phenomena in a particular social context (Bryman 2016; Creswell & Creswell 2017). Qualitative research is most preferred when researchers want to understand subjective perspectives regarding the phenomena under study (McGrath, Pamgren & Liljedah 2019). Flick (2018) found qualitative research to be important for developing rapport with participants and helping them feel free to share their experiences. Additionally, Kabir (2018) found qualitative data to be useful as it allows the researchers to take note of perceptions, emotions as well as feelings in the research process. Given the definitions, the qualitative design was suitable for this research because the researchers intended to investigate on the lived experiences of youths with disabilities. Semi-structured interviews were found to be suitable because they allowed the researchers to obtain data in the form of conversation, using both open ended and closed questions. The conversation revolved around the topic rather than following the rigid questions that characterise standard interviews. Focus group discussions were chosen because they encouraged reciprocal learning as the youths with disabilities engaged in discussion on specific topics. The researchers were able to get diverse views on specific topics on the sexuality of youths with disability.

Data from 20 semi-structured interviews, 2 focus group discussions, comprising 6 to 8 young participants with disabilities, were collected from Wards 6, 7 and 8 in Gweru. Five key informant interviews were conducted with representatives of state and non-state actors who are involved in provision of general services such as welfare and advocacy and sexual health-related services to youths with disabilities. Interviews were conducted in Shona and English; participants were asked to choose their language of preference.

Table 1 shows the socio-demographic characteristics of youths with disabilities who participated in the study such as names (pseudonyms), age, gender, and impairment type. Key informants were selected based on their practical experience in providing services such as case management, counselling, and justice and legal services to youths with disabilities. Seniority was considered and all key informants had more than 5 years of work experience. Youths are usually people aged 15 years to 35 years (Africa Youth Charter 2006). However, for the purpose of this research, only youths aged 18 years to 35 years were considered for participation because they are considered legally competent to give consent. Participants were aged between 18 years and 24 years. Five of these participants were aged between 25 years and 30 year, and 7 participants were in the age group 31 years to 35 years. The primary researcher worked with an organisation for people with disabilities to recruit participants. WhatsApp messages about the study were circulated in WhatsApp groups for youths with disabilities. The participants referred other youths with disabilities until the desired sample size was reached.

**TABLE 1:** Showing socio-demographic characteristics of participants.

Name (Pseudonyms)	Age (years)	Gender	Impairment type
Taona	22	Male	Club foot
Primrose	32	Female	Short right limb and albinism
Nashy	24	Female	Short lower left limb
Trickstar	30	Female	Stroke
Chichie	35	Female	Short right limb
Thomas	18	Male	Epilepsy
Tintin	27	Female	Cleft foot
Teekay	18	Male	Mobility impairment
Shamiso	25	Female	Short left arm and albinism
Tinaye	24	Female	Epilepsy and albinism
Jones	35	Male	Right arm amputee and albinism
Memory	34	Female	Epilepsy
Susan	35	Female	Club foot
Phineas	24	Male	Albinism
Peter	25	Male	Epilepsy and cerebral palsy
Junior	26	Male	Epilepsy
Blessing	34	Female	Right leg amputee
Simplex	33	Male	Left arm amputee
Ben	19	Male	Short left arm
Lucas	21	Male	Short right arm

According to the 2022 Population and Housing Census, the Midlands province has a prevalence of functional difficulty of 10.3%, higher than the national prevalence of 9.2%. These statistics influenced the primary researcher’s decision to conduct the study in the Midlands province. Gweru was chosen because it has the highest population in the Midlands province, with a population of 300 000, of which the majority are economically disadvantaged and vulnerable (Matsa et al. 2021).

The study wards were selected through purposive sampling, as these are the oldest locations in Gweru, which are densely populated. The researchers saw a higher likelihood of getting the required number of participants in these highly populated locations. Etikan et al. (2016) found that purposive sampling involves the inclusion of a participant or research site because of certain attributes that they have, which are important to the research.

## Data analysis

This study utilised thematic analysis to analyse the data from the semi-structured interviews and focus group discussions. Thematic analysis is a method of extracting meaning from data, and it involves identifying and recording themes, as well as social patterns that emerge from the data (Javadi & Zarea 2016).

The first step was to read the interview transcripts over and over, in-order to be familiar with the data. Notes were written on the early impression found from the data. The second step was organising the data in a systematic and meaningful way through coding. Data were reduced into smaller chunks. Theoretical thematic analysis was adopted as researchers were more concerned about addressing specific research questions; data were analysed with the research questions in mind. Open coding was used; the researchers did not have any preset codes. The codes were developed during the coding process. The researchers coded the same transcript separately, coding every relevant part of the transcript that addressed the research questions. After the researchers finished coding, they discussed, compared the codes, and modified some; this was done to all the other transcripts. The coding was done manually.

The third step was searching for themes. The researchers closely examined the codes to see how they fitted into themes. Other codes were combined to make a theme. After this process, the codes were organised into themes about perceptions of youths with disabilities in family contexts and their assumptions of sexuality. The fourth step involved reviewing and modifying the identified themes. The researchers combined all the data that were related to each theme. The themes were reviewed again to ensure that they were distinct and coherent. Themes were examined for overlaps; where such overlaps were observed, the themes were combined. The next step was defining the themes and at this stage the themes were refined and examined to identify their meanings. The relationship of the subthemes to the theme was also examined. The final step was the write-up.

The study utilised intersectionality theory to explain perceptions of youths with disabilities on family contexts and the assumptions of their sexuality. The term intersectionality was first coined by Kimberle Crenshaw (1989). The concept was first used among African-American feminists, disability feminists and Marxist feminists, but has since become a common concept when conceptualising issues of inequality and injustice. The theory is quite useful as it attempts to understand how various identity axes including but not limited to disability, gender, sexual orientation, culture, and ethnicity among others, can enhance or prevent varying forms of advantage or disadvantages. Intersectionality links various intertwined social categories, power dynamics, social inequalities, and various social contexts. It is linked to a very long history of black and third world feminism. Intersectionality has come to be known as the framework for understanding notions of difference, and the resistance of essentialism of difference (Hankivsky 2014; Hill Collins & Bilge 2016). Using the intersectionality theory, the researchers were able to understand the inter-link of disability, culture, gender, and social and familial perceptions that deny sexuality education and opportunities for dating and marriage to youths with disabilities.

## Ethical considerations

An application for full ethical approval was made to the North-West University Human Social Sciences Research Ethics Committee (NWU-HSS-REC) and ethics consent was received on 25 May 2022. The ethics approval number is NWU-01155-22-A7. Gatekeepers approvals were sought from: the City of Gweru; Office of the District Development Coordinator; Zimbabwe Republic Police; and the relevant Ward Councillors. Andoh-Arthur (2019) confirms that gatekeepers are important intermediaries for accessing research sites and participants. They have the power to allow or deny access to researchers.

### Confidentiality and anonymity

The research adhered to the principles of confidentiality and anonymity. To ensure privacy and confidentiality, no other person, except the interviewees, was allowed during semi-structured interviews and during focus group discussions. Pseudonyms were used to protect the identity of study participants. The researchers maintained strict security of the data to protect research participants, as well as state and non-state institutions. The researchers kept all personal data, field notes, and transcriptions on a password protected computer and hard copies in a locked cabinet.

### Informed consent

The primary researcher explained the purpose of the study to the participants, and participants were encouraged to ask any questions about the study. Study participants were informed that their participation was voluntary, and that they could withdraw from the study if they were no longer willing to continue. Participants were asked to read and sign a consent form in the language of their choice, or in cases where participants could not read or write, a witness would read it for them.

### Findings

This section presents and discusses findings on parental over-protection of youths with disabilities, locking up of youths with disability, denying them sexuality education, gender disability, and sexuality and the abstinence thrust of sexuality education.

### Over-protection by family

The study established that some youths with disabilities were overprotected by their parents and guardians. This restricted them from exploring relationships and dating. The youths with disabilities were not allowed to go to functions or public spaces. These restrictions closed sexuality spaces for them since it is in these public places that they could meet potential partners. Over-protection reduced their confidence and lowered their self-esteem. This is shown in the following account:

‘My parents were over protecting me, I was not allowed to go to a boarding school. They did not want me to go far away from them. This over-protection impacted me negatively. It hindered me from experiencing and experimenting on relationships as a teenager. I was not allowed to go out with other kids. They were worried that I might get injured or sexually abused. In the process, they were closing the opportunities to get into relationships. I grew up as a person who cannot go to gatherings or any place where there were more people. I have some fear, and this results from my upbringing. Sometimes over-protection results in future problems.’ (30 years old, female, stroke)

Because of parental overprotection, Trickstar did not have ordinary teenage experiences as her peers. She had less knowledge about relationships because of lack of exposure. Although her parents had good intentions, such overprotection resulted in challenges related to socialising with peers:

‘Our parents sometimes over protect and spoil us. They are not comfortable when we are away from them. They have a feeling that we will be ill-treated. I was staying with my grandmother. When she died, my mother had to resign from her job to take care of me. She was worried that I might be sexually abused. I introduced her to my boyfriend, but she was worried, and she told me she does not want me to get married because men cheat on their wives and she does not want me to be disappointed. Our families are overprotective. However, it becomes a disadvantage to the young women with disabilities who are denied a chance to explore life and date. They forget that one day they will die and there will be no one to take care of us. If one gets married, their spouse and children will take care of them.’ (24 years old, female, short lower left limb impairment)

From the participant’s narrative, her mother still considers her to be a child who cannot make her own decisions. Although she has a boyfriend, her mother does not want her to get married.

### Youths with disabilities locked up because of societal discrimination

The following excerpts illustrate how locking up youths with disabilities hinders them from experiencing dating and marriage. They are denied opportunities for mutual learning through peer association. Families with members who have disabilities face societal discrimination resulting in some parents and guardians resorting to locking up and hiding youths with disabilities because of fear of stigma (Goffman in Clair 2018):

‘Parents keep their children with disabilities indoors in fear of cultural stigma.’ (27 years old, female, cleft foot)

‘I was not allowed to go to certain functions where the other family members were going. I think my family would be discriminated against if people saw that they had a disabled child. Our culture is not inclusive.’ (22 years old, male, amputee)

Contrary to Trickstar’s account and the 24-year-old female focus group participant who were overprotected by parents because of safety concerns, the account of Taona and the 27-year-old female focus group participant shows how their families were also protecting themselves from disability stigma through locking up youth with disabilities. This disability-related stigma was not only experienced by the youth with disabilities but extended to their families too:

‘Some young people with disabilities are discriminated against in their homes and in the community. Most of them spend their entire childhood locked indoors; therefore, they lack exposure and voice.’ (39 years old, male, key informant)

Some youths with disability are rendered inarticulate when it comes to issues of sexuality because of being under exposed. This also exposes such youth to sexual violence in the family as youth who are locked indoors cannot report cases of sexual violence or seek SRH and other support services.

### Most youths with disabilities are not offered sexuality education

Most participants reported not being offered sexuality education by their parents or guardians. This was linked to socio-cultural norms that regard sexuality as taboo and inappropriate subject in the family. Because people with disabilities were not expected to get married and have children, their families were reluctant to teach them about sexuality. In some families, all the children did not receive sexuality education including those who do not have disabilities. The following accounts demonstrate participants’ experiences with sexuality education in the family:

‘I was never taught about sexuality due to our Shona culture. It is taboo among the Shona for a parent to talk about sex with children. Uncles and aunts are the ones who can do this but, in our case, our aunts and uncles do not stay in Gweru. They rarely visit us. We only meet at family gatherings, and usually there will be no time.’ (18 years old, male, epileptic)

‘My uncle used to teach me about becoming a man. He taught me to be responsible and avoid pre-marital sex.’ (21 years old, male, short left limb)

‘I got this information from my aunts and sisters, and not from my parents. When my aunts visited us, they would teach me about relationships. My aunts would make time to speak to me privately about sexuality every time they visited.’ (35 years old, female, short limb)

Lucas and Chichie’s narratives confirm the socio-cultural norms that govern sexual communication among the Shona people. Parents are not culturally equipped to teach their children about sexuality. They delegate this role to aunts and uncles as prescribed by the Shona culture. Uncles and aunts are the support system for sexuality issues and social values. The findings reveal how urbanisation has disturbed the structures of traditional extended families. The traditional family set up has undergone changes in Zimbabwe; it is now difficult to find time and have sexuality discussions with uncles and aunts who may be staying in other, families are being forced to adapt to change (Nyanungo 2018). The study found participants whose parents subscribed to the socio-cultural myth of sexuality as taboo to be disadvantaged as they were deprived of sexuality education.

### Gender, disability, and sexuality

Gender is an important consideration in the provision of sexuality education. Male youth received sexuality education from their uncles or fathers. On the other hand, female youth were taught about sexuality by their mothers or aunts. It was not common for fathers to impart sexuality education to their daughters as illustrated in the following accounts:

‘I got information from my father when I wanted to leave home to look for a job. My father talked to me about being careful with relationships and the importance of remaining focused. He also told me that when I find a boyfriend, I should come with him home, so that the family can see him and he could be held accountable when I am impregnated.’ (35 years old, female, club foot)

‘My uncle used to teach me about sexuality and becoming a man he taught me that I would experience physical changes in my body. He also taught me to be responsible and avoid pre-marital sex.’ (21 years old, male, short left limb)

‘In the African context sexuality is not discussed in the family. I was raised by my mother, my father died when I was still young. Maybe due to gender issues my mother failed to teach me about sexuality. Maybe if it were my father, he would have taught me about sexuality, or my uncle. Unfortunately, we do not have good relations with my uncles.’ (26 years old, male, epileptic and cerebral palsy)

Susan’s account shows that even fathers can give sexuality education to their daughters. This contrasts Lucas’ narrative which reveals the standard practice among the Shona assigns sexuality education to uncles and aunts. Sexuality is generally regarded as a taboo topic, especially when a male parent discusses it with a female child, and when a female parent discusses sexuality with a male child. Lucas’s account reflects the standard practice according to Shona culture, which assigns the teaching of sexuality education to paternal uncles and aunties. Junior’s narrative shows how women are not culturally capacitated to teach sexuality education to their sons. A study by Rugoho et al. (2020) reported some differences in how families offered sexuality education to disabled youth. Youth males were expected to get married 1 day; therefore, they were taught about sexuality more comprehensively than their female counterparts who were not expected to marry.

Families have low expectations for disabled youth and this becomes a barrier to sexual education. They are also incorrectly assumed to be asexual and therefore they are excluded from sexuality education. Although sexuality is considered as taboo subject by parents in general, it is considered unthinkable to discuss sexuality of children with disabilities. The following extract illustrates the idea of increased taboo of sexual communications with disabled youth:

‘As you know, in a Shona family like ours it is taboo for a parent to talk about sexuality in general, and even greater taboo to engage such discussions with a disabled youth. So, I was not taught about sexuality by my parents.’ (34 years old, female, amputee)

Socio-cultural beliefs about disability and sexuality inform the perceptions of the taboo nature of sexuality. There are myths and misperceptions that people with disabilities are not able to control their sexual drive, and therefore they should not be introduced to sex or involved in discussions about sexuality. People with disabilities are also assumed to be less interested in sex, and therefore some parents do not see the need for educating them on sexuality. In contexts where these beliefs are prevalent, disabled youth are deprived of sexuality education.

### Sexuality education that emphasises abstinence

Data from this study revealed that most disabled youth who managed to get sexuality education in their families were mainly taught about abstinence. The emphasis on abstinence is in line with the cultural values in the study area, which shun pre-marital sex:

‘When we were growing up it was just taboo to talk to a teenager about using a condom. The key word that we were taught regarding sex was “abstain”.’ (34 years old, female, amputee)

‘Sexuality education offered in schools is one sided as it focuses on abstinence alone without emphasizing on the use of condoms. Young people do not take calls to abstain seriously as they think it is not practical. Therefore, there is need to emphasize on the use of protection when indulging in sexual intercourse.’ (24 years old, male, albinism)

Although abstinence is compatible with cultural values, sexuality education that wholly focusses on abstinence did not give disabled youth enough information and the youth perceived it to be a bit outdated. Youth also needed to be taught about other methods of safe sex such as using contraception as some youth still indulged in sex despite calls for abstinence.

### Challenging misconceptions on sexuality of people with disabilities through teenage dating and parenthood

Although disabled youth have been assumed to be asexual and unsuitable as partners, some participants revealed that they started dating when they were teenagers. The following excerpts illustrate the experience of disabled youth in teenage relationships:

‘I got into my first relationship when I was in form three so I was 16 years old I kept it a secret because my family would not accept that.’ (34 years old, female, amputee)

‘When I started dating, I was 16, you would be willing to experience how it is to be in love and experience new things just like other girls.’ (24 years old, female, epileptic and albinism)

The excerpts show that youths with disabilities start dating earlier just like their peers without disabilities. They also experiment just like their peers without disabilities. Youths with disabilities are not passive recipients of negative stigma but they secretly engage in relationships to experience what other youth without disabilities experience.

For most participants who had experienced motherhood and fatherhood, these parental experiences are silent ways of challenging the misconceptions about disability and sexuality:

‘When I entered a relationship that resulted in the birth of my child, there were some challenges. The father of the child was thinking that I was going to give birth to a child with a disability and some blemishes. Later, he realised that the person he impregnated is just the same as anyone who does not have a disability, after I delivered a healthy child without any disability.’ (30 years old, female, stroke)

‘My parents never wanted me to be in a relationship, they never imagined me getting married or having a child due to my disability. Although they wanted me to be happy, they were aware of the stigma associated with dating and marrying a person with a disability in our society. However, I know what I want in life, so I secretly dated my boyfriend. We would meet at school and other secret places. I secretly eloped and started staying with him. There was a challenge with getting accepted by his family. It is very difficult to be accepted by the family of a non-disabled partner. My boyfriend’s relatives were against the relationship. I was ready to fight for our marriage. I wanted to be a mother like other women. Choosing a disabled wife was not acceptable to the family. Most people think being an albino is a curse. They assume that if you marry an albino, you will give birth to children with albinism, but that is a lie. The father of my child does not have albinism and my child does not have albinism.’ (32 years old, female, short right limb and albinism)

These narratives demonstrate the agency of disabled youths with disabilities in resisting negative perceptions of their families and society regarding dating and marriage. All participants who had given birth and had children did not a have disabilities. This challenged the myth that disabilities can be passed on to children from parents. This lessened the stigma and the discrimination against women with disabilities. Giving birth marked a transition to motherhood, and this brought a higher status to young women with disabilities. Experiences of motherhood were considered by the participants as passive forms of resistance to the negative perceptions about people with disabilities that view the people with disabilities as lacking in sexuality.

## Discussion

This article examined the perceptions of youths with disabilities about their sexuality, and impact of socio-cultural assumptions on disability and sexuality. The findings convey five themes that form different forms of social closure, which can be summed as follows:

Firstly, the theme of social protection that families enforce in the activities of the youths with disabilities, and this may be justified to the extent that it reflects parental care for their offspring, particularly to protect them from being socially or sexually abused. Such an ethic does not form a closure. However, when conceived as over-protection, the sexual life of such youth is closed off, remains off-limits, or is considered taboo. Confining youths with disabilities to their homes is in effect locking them off social life (Peairson et al. 2014) and does not assist in breaking down the barriers of social shame that arise from the societal stigma of disability (Chappell 2016; Frappier 2021; Rugoho et al. 2020). This is one of the points of intersectionality of oppression for youth with disabilities but is limited to parental overprotection rather than parental care.

Such overprotection is also demeaning to their sexual autonomy, which is under threat (Braathen, Rohleder & Azalde 2017; Rugoho & Maphosa 2017). One dire consequence of such overprotection is that when the parents pass away, the youths with disabilities are rendered helpless because of dependence on over-caring parents. Parental assumptions of the immaturity of their children with disabilities perpetuates such helplessness (Guzman & Platero 2012; Smirnova & Verbilovich 2020) or reduce such youth to the status of children (Chappell 2016) and thus need to be closely monitored (Santos & Santos 2016). Parental care is important but needs to be directed towards allowing the autonomy of such youth as much as possible. Beyond parental care, such youth need to create other worlds of care, connections, and social worlds that can empower them more sustainably. Such youth need intersectional socialisation to widen the net of positive forces that can empower their lives. Overprotection by families opens to possible abuse within the family, as limitations are set on extra-family interactions because of social stigma. Such limitations form another point of social oppression for young disabled people. If they are allowed a greater network of relations and interactions, their social field would be open to more possible support mechanisms, while institutional structures need to be working on civic education to change societal mindsets on sexuality and disability.

Secondly, the notion of public social space is central to disabled youth and their identity formation. Such a space is cordoned off, understandably because of parental fears of abuse that youth may get exposed to. The result is an asocial upbringing that forecloses actions that can be useful for such youth, including a wide variety of civic interactions possible as in many societies where the disabled are empowered. Social stigma against the disabled casts powerful negative taboos against the latter and needs to be worked upon both within the family and in society at large, for more effective and positive socialisation for such youth. Both a constrained public space and negative social stigma delimits the existence of disabled youth and conveys a structural constraint for the latter.

Education is another significant theme that conveys the possibility of intervening in intersectional oppression and emerges from the views of disabled youth. Because of the debilitating effects of urbanisation on traditional extended family systems and their protective mechanisms for the disabled (aunts, uncles, and peers chipping in to assist with sex education), and with some parents abiding by the norm that ‘sex talk is taboo’, a mixed picture arises. Support may emerge in the form of more liberated fathers talking to their daughters or grandmothers advising grandchildren. Yet, social taboos on sexual matters may be overriding and hold back on such more open forms of discourse to emerge and overtake such views relating to sex as taboo. Education is thus necessary, both societal and in the family, to tackle both the stigma attached to these matters and to reinforce more positive forms of socialisation, both in civil society and in the family. Sexual and reproductive health services would have to comply with such reinforcements, and schools, media, government, and non-governmental organisations may need to work together to enhance such education. Contextual education and policy must be emphasised, so that both parental care and youth development are catered for without foregoing the sexual autonomy and identity of the youth.

Taboo topics must be carefully approached in educational institutions, programmes, services to gain maximum benefit for both families and disabled youth. Sex talk across sexes between son and mother, or father and daughter, is usually taboo and not always possible (DAWN 2020; Frappier 2021; Williams et al. 2014). But if it emerges it needs to be encouraged, and if there is resistance to such empowerment, other empowerment mechanisms need to be explored.

Thirdly, sex abstinence may not always be possible, but where it is done with the care of disabled youth by parents advising sex after marriage, such advice may need to be seen as useful particularly as these youth face the diverse dangers of abuse, Still, the overriding emphasis on sex abstinence as a law or canon, forms another closure in the network of interpellations and intersectional oppressions, as community expectations confirm (Rugoho et al. 2020). Alternatives to abstinence need to be added to educational and programmatic structures that relate to disability and sexuality issues. Struggles against more dominant views of sex, disability, and the expansion of the social life of the disabled remain, but wherever the space allows for intervention, that space needs to be used to improve the discourse on it, to lend further to the improvement of matters relating to sexuality and disability.

Lastly, the agency of disabled youth in challenging negative socio-cultural and familial perceptions regarding their sexuality was explored. Through dating, marriage, and childbirth experiences, disabled youth challenge myths about their asexuality, unsuitability for marriage, and inability to bear children. Participants demonstrated passive power by refusing to accept the asexuality label and indulging in teenage relationships. This finding resonates with a study conducted by Karimu (2017), in Ghana, where she found that even in contexts where sexual activities and conversations on sexuality were forbidden, young people with disabilities always found ways of expressing their sexuality and dating. Although families and society did not recognise the sexuality of disabled youth, a study conducted in Nepal by Devkota et al. (2018) has revealed that people with disabilities have the same sex drive as their peers without disabilities. This motivation to oppose negative stereotypes of society regarding sexuality of disabled youth is in line with Slater et al. (2018) study, which revealed that young women with disabilities were determined to refute the disability stereotypes that portray them as unattractive.

## Conclusion

The study concludes that most of the sampled youths with disabilities were given very little, if at all, sexuality education in their families, because of the negative perceptions of parents and the community. People with disabilities are assumed to be ambiguously, asexual and/or hypersexual. Therefore, the sexuality of people with disabilities is not discussed in families. Some parents feel embarrassed and unprepared to discuss sexuality with youth with disabilities and usually delegate this duty to uncles and aunts. Some participants reported that sexuality education offered by parents and guardians emphasised abstinence only, which the youth perceived as lacking. The study recommends appropriate programmes for training all carers, biological or institutional, on the sexuality of people with disabilities. Negative beliefs about the sexuality of people with disabilities need to be countered with more positive treatment in all civic, political, and social encounters in both the public and family realm, with the support of the media to implement this and promote the sexual agency of youths with disabilities. The study further recommends the development of a manual for out of school sexuality education that is accessible to the public and in local languages and use of media platforms to convey such messages.
